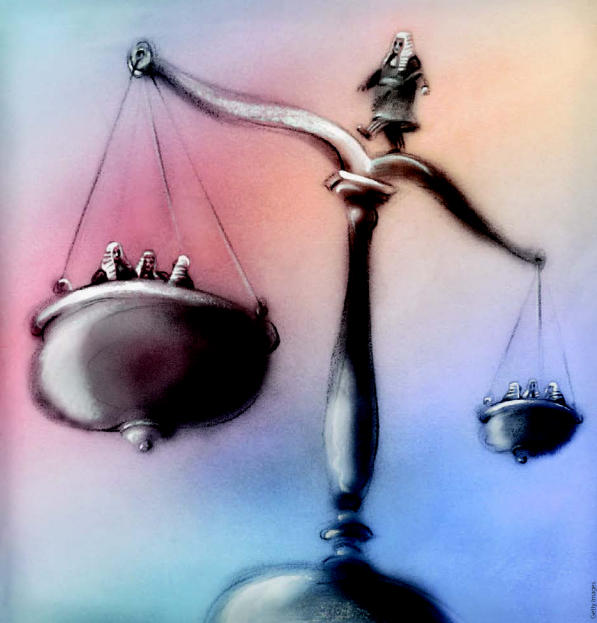# A Changing Climate of Litigation

**DOI:** 10.1289/ehp.115-a204

**Published:** 2007-04

**Authors:** Richard Dahl

Frustrated by perceived federal reticence to act on the growing scientific evidence of climate change, state governments and environmentalists are increasingly turning their attention to the courts. Broad consensus has developed about the reality and seriousness of global warming, but neither the Bush administration nor Congress has yet responded with meaningful action. The result is a situation that is ripe for litigation. Plaintiffs have emerged, suing corporations on the grounds that their greenhouse gas emissions are causing undue harm and suing governments for failing to regulate the corporations. In addition, industry has responded with countersuits of its own.

To date, plaintiffs in climate change lawsuits haven’t scored any big victories, and prospects for the future are unclear. In fact, even its strongest supporters admit that litigation, by its nature, provides a piecemeal approach to dealing with a problem that should be addressed broadly by the legislative branch. “I’m the first person to say this is not a very effective means of addressing the problem,” says Sierra Club senior attorney David Bookbinder, who has been on several plaintiff legal teams involving global warming. “But it’s the only one we’ve got.”

“There’s a period where there’s an accumulation of scientific evidence, yet the cases don’t succeed. But then the gradual accumulation becomes overwhelming, consensus changes, and the law follows.”

– Joseph Smith University of Adelaide

However, there’s also a belief by its adherents that litigation can ultimately play a key role in shaping broader public policy. Australian lawyer Joseph Smith, a researcher at the University of Adelaide, has been studying the emergence of climate change litigation in the United States, Australia, and elsewhere in the world, and he thinks it may follow a pattern similar to those of tobacco, asbestos, and other “toxic tort” categories, in which personal injury is caused by exposure to a hazardous agent. “There’s a period where there’s an accumulation of scientific evidence, yet the cases don’t succeed,” he says. “But then the gradual accumulation becomes overwhelming, consensus changes, and the law follows. I don’t think this is going to go away.”

## Supreme Court in the Spotlight

To a large extent, the future of climate change litigation—in the United States, at least—now rests in the hands of the U.S. Supreme Court. In November 2006, justices heard lawyers argue *Massachusetts et al. v. Environmental Protection Agency et al.*, the first climate change case that the nation’s top court has heard. In that case, Massachusetts, along with 11 other states and several cities and nonprofit organizations, sued the EPA in an effort to compel it to regulate carbon dioxide emissions from motor vehicles under the Clean Air Act. Then–EPA general counsel Robert E. Fabricant had concluded in an 8 September 2003 memorandum that the agency doesn’t have the authority to regulate any greenhouse gases under the act—and that furthermore, even if it did have the authority it would choose not to exercise it.

The EPA’s stance that it lacks the authority to control *any* greenhouse gases means that the outcome of *Massachusetts v. EPA* will apparently affect stationary sources as well as motorized vehicles. Justices will be issuing their opinion—or opinions—by June 2007. And nobody’s making any predictions about how it might turn out. At the oral argument, the justices focused much of their attention on the issue of “standing,” which refers to the requirement that plaintiffs show they have suffered an injury that is traceable to the defendant’s action.

The justices’ focus on standing is an illustration of the difficulties that plaintiffs have in making winnable legal arguments in climate change cases. Washington, DC, lawyer Russell S. Frye represents the CO2 Litigation Group, an umbrella of several business trade associations that supports the EPA in the case. He says that pinning the blame in climate change cases is difficult to do. “When you’re talking about how the United States in particular ought to respond to a concern that’s raised by emissions and developments throughout the world and not just the United States, it’s hard for individual states or environmental groups to show that the relief they’re seeking will redress their injury,” he says.

That is, Frye says, climate change is caused by an atmospheric layer to which the entire world is contributing. If a defendant is forced to reduce emissions, the harm will be reduced only slightly. “Everyone is saying this is a really serious problem that needs action, and yet the action they’re taking, even if successful, would only impact a small percentage of carbon dioxide emissions,” he says.

Furthermore, he says, climate change plaintiffs “are raising questions that are not for the court to resolve. Certainly, I don’t think the founding fathers thought that the judicial branch was where policies like this should be made.”

But plaintiffs’ lawyers in these cases say they’ve brought the actions because they were the only available alternative. “We started these cases in the darkest days of the Bush administration’s rejection of doing anything,” says David Doniger, policy director of the Natural Resources Defense Council’s Climate Center. “We’re pursuing the litigation for the simple reason that you pursue all avenues.”

“I’m the first person to say this is not a very effective means of addressing the problem. But it’s the only one we’ve got.”

– David Bookbinder Sierra Club

In fact, climate change litigation may be more widespread than most people realize. Last fall, Georgetown University Law Center fellow Justin R. Pidot surveyed the existing litigation in the United States and found that *Massachusetts v. EPA* was one of 16 pending lawsuits involving climate change. He found the suits evenly distributed among four basic categories: 1) Clean Air Act suits, such as *Massachusetts v. EPA*; 2) National Environmental Policy Act suits, which claim that government agencies must include the consequences of climate change when they measure the environmental impacts of projects they fund or license (these lawsuits have been the most successful); 3) preemption suits, brought by industry plaintiffs against states such as California with tougher emissions standards than the federal ones; and 4) nuisance suits, which contend that contributors to climate change are creating a common-law nuisance.

## A Battleground in California

All eyes in the world of climate change litigation are on the U.S. Supreme Court, but a great many of them are also on California, where attorney general Jerry Brown and six auto manufacturers are waging a legal war that’s attracted international attention. The federal Clean Air Act includes a provision that allows California to set its own carbon dioxide emission standards for motor vehicles and also allows other states to adopt California’s standards if they wish. In 2004, California lawmakers decided to proceed on setting the state’s own standards with a requirement that automakers begin reducing emissions starting in 2009, and 10 states have lined up to follow them. The automakers objected, arguing that the California law is preempted by the federal Energy Policy and Conservation Act, which created the corporate average fuel economy (CAFE) standards. They filed suit in 2005 (*Central Valley Chrysler-Jeep v. Witherspoon*).

Then–attorney general Bill Lockyear responded in September 2006 by filing a suit against the automakers (*California v. General Motors*) on the theory that they have created a public nuisance with the greenhouse gas emissions that their products create in California. Brown, who was elected in fall 2006, has pledged to carry on the fight.

There has been one other large-scale climate change nuisance suit, *Connecticut v. American Electric Power Company, Inc.* The plaintiffs in this case—eight states, the city of New York, and three land trusts—were not successful. They sought an injunction to stop the five biggest U.S. carbon dioxide emitters (American Electric Power Company, Southern Company, the Tennessee Valley Authority, Xcel Energy, and Cinergy Corporation) from conducting business as usual. Together, these emitters send about 650 million tons of the gas into the atmosphere each year.

The plaintiffs asked a federal court in New York to issue an abatement order to reduce the emissions. However, a judge dismissed the case in 2005, saying that public policy about greenhouse gas emissions was a “political question” that needed to be answered legislatively. The plaintiffs appealed to the U.S. Second Circuit Court of Appeals, where the case has been fully briefed and argued, and an outcome is pending.

Ken Alex, supervising deputy attorney general in the California Attorney General’s environmental section, believes that the California nuisance suit is different, however, because it seeks damages from the automakers. “The types of damages include things like the millions of dollars the state is spending to address the impacts of global warming,” he says. “There are concrete impacts already. For instance, our flood control system was built with the idea that it control five-hundred-year flood events, but now those flood events are more like fifty-year events because of the earlier and more substantial snow runoff in the Sierras. The value of the flood control system has taken an economic hit, and it needs to be rebuilt.”

But Theodore J. Boutrous, the Los Angeles–based lawyer for the automakers, counters that Brown’s office is out of its league in trying to impose itself on his clients. “These global warming issues are complex, delicate, political, scientific issues that need to be resolved in a comprehensive, careful way through the political process as opposed to [in the] courts, which decide things on an ad hoc basis that doesn’t allow for the kind of analysis that is required in this area,” he says. Furthermore, he also questions the firmness of the state’s legal ground in seeking to collect damages for auto emissions when the state operates a huge fleet of vehicles that itself is contributing to the problem. (California governor Arnold Schwarzenegger pledged in 2004 to gradually replace the state’s fleet, currently numbered at 37,000 vehicles, with hydrogen-powered vehicles.)

The outcome in both California cases—as well as in other climate change lawsuits—depends on how the Supreme Court rules. A plaintiff victory presumably would eliminate the nuisance suit against the auto manufacturers as well as the pending suit in the Second Circuit Court of Appeals. But as Bookbinder points out, a resounding loss may not actually be a bad thing. “If we lose everything, then it’s up to Congress—and I can live with that,” he says. “Pressure is mounting for them to address this issue.”

## The Global Perspective

Elsewhere around the world, there also have been developments in climate change litigation. Smith and a University of Adelaide colleague, physician David Shearman, recently co-authored a book, *Climate Change Litigation*, that examines the issue in more of a worldwide perspective, although they focus on the United States and Australia for examples. Shearman is a longtime member of the volunteer group Doctors for the Environment, Australia, and a contributor to the health sections of the 2001 and 2007 assessments of the Intergovernmental Panel on Climate Change, while Smith applied legal analyses to examine the kinds of legal responses that hold promise.

“[Climate change plaintiffs] are raising questions that are not for the court to resolve. Certainly, I don’t think the founding fathers thought that the judicial branch was where policies like this should be made.”

– Russell S. Frye C02 Litigation Group

To Shearman, the key to making effective legal arguments about global warming rests in the scientific data about the health effects. As an example, he cites the 2003 European heat wave, which resulted in an estimated 22,000 to 45,000 “excessive deaths” (the number above the normal death totals for the period). That kind of heat is supposed to happen every 50 to 100 years in Europe, he says, but “according to the probabilities of climate change, by 2050 such a heat wave will occur every fourth year in France.”

Smith and Shearman say Australia is similar to the United States at the moment—both are countries with conservative national governments that are loath to regulate industry. But Smith points out that even in Australia there’s been progress. In November 2006, the New South Wales Land and Mining Court handed down a decision in *Gray v. The Minister for Planning and ORS* that requires that government agencies now consider the effects of greenhouse gas emissions involved in all new building projects and land development.

Around the world, Smith says there have been successful lawsuits against U.S. companies using human rights arguments against a nation that currently emits the most greenhouse gases per capita but that is not a Kyoto Protocol signatory. Most recently, in March 2007, representatives of the indigenous Inuit people argued before the Inter-American Commission on Human Rights that the United States is violating their rights by causing climate change that threatens their traditional way of life—melting sea ice upon which their villages are built and threatening species upon which they depend for survival. In Europe, meanwhile, the European Union has created a court to hear cases against member states if they don’t comply with EU emission standards.

Environmentalists and lawyers who have studied climate change litigation agree that the ideal venue for change would be some kind of world court. Smith says that even the Kyoto Protocol lacks an international judicial forum. He says he has done extensive research on the role that international law might play in reducing global warming, “and it comes to a dead end. It seems that, in the end, it needs to be national laws to get things done.”

“In a nutshell,” Smith concludes, “each discipline and field can make a contribution. But none of them is sufficient on its own to really carry the weight. It’s got to be everyone working together at both the individual and international levels to deal with it. There’s no one solution.”

## Figures and Tables

**Figure:**